# Scanning Strategies in Laser Surface Texturing: A Review

**DOI:** 10.3390/mi14061241

**Published:** 2023-06-12

**Authors:** Denys Moskal, Jiří Martan, Milan Honner

**Affiliations:** New Technologies Research Centre (NTC), University of West Bohemia, Univerzitni 8, 30100 Plzeň, Czech Republic; moskal@ntc.zcu.cz (D.M.); honner@ntc.zcu.cz (M.H.)

**Keywords:** laser machining, laser ablation, processing rate, scanning techniques, heat accumulation, plasma shielding

## Abstract

Laser surface texturing (LST) is one of the most promising technologies for controllable surface structuring and the acquisition of specific physical surface properties needed in functional surfaces. The quality and processing rate of the laser surface texturing strongly depend on the correct choice of a scanning strategy. In this paper, a comparative review of the classical and recently developed scanning strategies of laser surface texturing is presented. The main attention is paid to maximal processing rate, precision and existing physical limitations. Possible ways of further development of the laser scanning strategies are proposed.

## 1. Introduction

Laser pulse surface ablation leads to the formation of a crater with a depth of several nanometres up to micrometres. The application of consecutive laser pulses with sequential laser beam movement facilitates the creation of regular laser-formed surface structures, from simple periodical dimples or segments up to polygonal or hierarchical structures [1,2,3,4]. Such a process of periodical objects laser formation is named laser surface texturing (LST), and it has a wide range of applications: in tribology, material engineering, wettability, brazing, medicine, optics and other areas [5,6,7,8]. The development of laser surface scanning techniques is needed for increasing the processing rate of LST. Involving the fastest scanning techniques, for example, the application of polygon scanners, acousto-optic beam deflection or sample rotation, has several limitations, such as dead processing time, low deflection angle, sample geometry specification and others. At higher laser surface scanning speeds, it becomes more and more difficult to maintain the high precision of LST, due to the synchronization loop between the laser and the scanning system [9,10,11]. The application of high-frequency lasers with ultrashort laser pulses meets the physical limitations of LST, such as heat accumulation between laser pulses and ablated plasma shielding effects [12,13,14,15,16]. The speed limitation for the application of high-frequency lasers becomes important for texturing of 3D bent surfaces [17,18]. There are alternative techniques, such as using low-frequency lasers with high pulse energy for processing entire wide areas at once with a low-speed laser beam or sample movement [19,20,21,22,23,24]. For such a technique, plasma shielding and heat accumulation do not have such an important role, but on the other hand, the flexibility and scalability of the laser-processed area is limited by a spot size in comparison with scanning technologies.

The correct choice of an appropriate LST method needs to have an overview of the actual scanning techniques and their limitations. There are a lot of reviews for laser surface scanning strategies for selective melting in 3D printing technology [25,26,27]. However, there are no systematic reviews of the existing laser beam scanning strategies for LST with a comparative analysis of their scanning parameters, such as processing rate or maximal scanning speed. In this paper, such a review of the classical and recently developed laser surface scanning strategies with a comparative analysis of several scanning parameters of LST is presented. The scanning strategy is defined as a spatial and temporal arrangement of laser paths on the scanning surface, which can be applied in LST.

Different laser surface scanning strategies will be more suitable for different materials, especially considering the laser source limitations [13]. The resulting efficiency, roughness or minimal periodicity of LST structure are significantly influenced by the laser source used. There are a lot of other laser surface processing parameters which significantly affect initiated physical processes of material ablation, such as laser pulse fluence, duration, wavelength, polarization, air pressure or blowing speed or even pulse parity [28,29,30,31,32,33,34,35,36]. The formation of periodically distributed micro-objects on the laser surface is additionally affected by the limitations of the laser beam scanning system and desired structure of the laser-formed texture. The full description of all passible physical mechanisms, which affect the resulting surface structure and effectivity of laser surface ablation, goes far beyond one article. 

The goal of this paper is to describe existing strategies of laser surface texturing beam scanning techniques, which principally differ only in laser beam path ordering and filling arrangement. For that reason, in this review, only technical parameters of laser beam scanning strategies in LST were compared: processing rate, surface structures intervals, precision and scanning speed. The main physical limitations, which are mainly affected by the chosen scanning strategy of LST, are also discussed. 

## 2. Physical Limitations of Laser Surface Texturing

There are a lot of physical effects which are activated during the interaction of a laser pulse with the material, depending on the intensity and pulse duration of the laser pulses: from slight surface heating up to intense Coulomb explosion [37]. However, the main physical effects, which are affected by the chosen scanning strategy, are heat accumulation and plasma shielding effects. Although some scanning parameters exist, where optimal heat accumulation and even plasma shielding effects have a positive role in achieving higher efficiency or better quality of processed surface [38,39], generally, they play a negative role in the biggest cases [40,41,42]. In this section, the mentioned physical effects are discussed as principally influenced by the choice of the scanning strategy.

### 2.1. Plasma Shielding Effect

The ablation plasma plume spreads over the laser-processed surface, and the next laser pulses will be partially or fully blocked by the plume. The time of the plasma plume surface shielding depends on laser pulse parameters, such as pulse duration, pulse energy or focused spot size. Understanding the main principles of the ablation plasma plume evolution helps in the correct choice of an optimal scanning strategy for LST.

**Figure 1:**
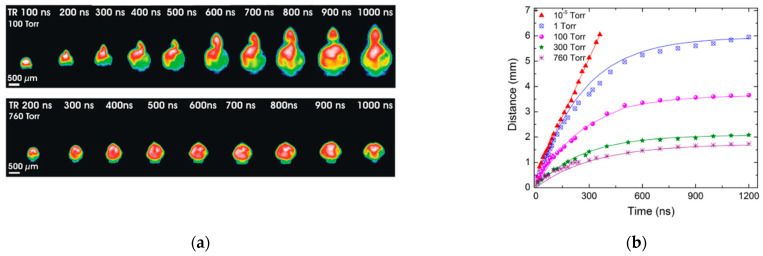
Explosion of ablation plasma plume: (**a**) plasma plume evolution [60,65]; (**b**) R-t plots obtained from ICCD images are given for various pressure levels [35].

The laser pulse absorption initiates the surface temperature reaching the region of overcritical fluid formation [37,43,44,45,46,47,48]. It was shown that ultrafast solid-to-liquid phase transitions already appear in the first few hundred femtoseconds [46]. Following this, material expansion is detected in 10–20 picoseconds after laser pulse absorption [46,47]. Exposed materials are able to achieve great speeds, more than 8–10 km/s [49,50,51,52]. The ablation plume is ejected at the highest speed in the phase explosion regime. The speed of the laser-ablated plume at the very start of the explosion can be described by a kinetic equation of adiabatic expansion [53]:

(1) $$v_{f}∼{\left(E/M\right)}^{1/2}$$

where M is the mass of ablated plume, and E is the total energy. In ambient gas, the free expansion of the ablated products will be changed by breaking the movement of the plasma plume with a generation of a frontal shock wave [49,53,54]. The following part of the ablated plume has slower medium-size clusters (up to 10,000 atoms) that have ejection velocities of less than 4 km/s and droplets that are slower than 3 km/s [49]. The pressure in the front shock wave is typically 100–200 atm and decays to close to ambient pressure in 100–200 ns [55]. In the latter time, the shock wave slows down to the speed of sound and travels forward as a sound wave [56]. The formation of this shock wave expanding starts in a short time period (0.2–0.5 ns), when the mass of the shock wave becomes comparable with the plume mass [57,58]. The following movement of the plasma plume in ambient gas can be described by the drag model [35,56,59]:

(2) $$R=R_{0}\left(1-exp\left(-\beta t\right)\right)$$

where R is the position of the plasma plume front during expansion, $R_{0}$ is the stopping distance of the plume, and β is the deceleration coefficient. The stopping position of the ablated plume at normal pressure of ambient gas above the scanned surface is about 1–2 mm and higher (Figure 1) [35,60]. The post-ablation products contain clusters and droplets, which continue to have high enough speeds ~100 m/s and inertial mass for achieving bigger distances above the laser-processed surface [61,62]. Such ablation products can cover several centimetres above the processed area for a long time, up to tens of microseconds [34,63]. Plasma and particle shielding effects significantly limit the laser repetition rate and effectiveness of laser surface processing [64].

The plasma plume transparency dynamic was studied in detail by J. König et al. [44]. The transparency of the plasma plume above the metallic target was measured by a probe laser beam, which was parallel to the sample surface. It was found that in the first moment of the plasma plume expansion, its transparency decreases down to 10%. The transparency of the plasma plume stays reduced by a certain value till 2–3 µs; during this time, the ablated material expands by hundreds of micrometres [35,56]. Such a highly optically dense plasma plume works as a shield above the processed surface for every subsequent laser pulse, if it comes before the plasma plume dissipates. Overcoming plasma shielding effects can be achieved via the application of a long enough time delay between laser pulses (MHz or lower frequencies), and then the plasma plume optical density becomes low enough before every next laser pulse. Such laser pulse frequencies are applicable for optical systems with laser beam scanning speeds up to 10–20 m/s, which are needed for optimal laser spots overlapping 30–50% [10,13].

An opposite way of overcoming the shielding effects can be achieved via the application of several laser pulses within a short time interval, shorter than that of a plasma plume, which will be expanded above the surface [15,66]. The plasma plume expanding cannot be avoided by the application laser pulse with a short time interval, down to 1–2 ps, but the energy conversion in such fast processes is able to change the ablation mechanism and suppress the shielding effects [15,66]. Such a shielding-suppressing regime is very dependent on processed materials, laser pulse intervals and even pairing of the laser pulses [28]. The frequency of laser pulses for such a mechanism of plasma plume suppression should be in the hundreds of GHz or in THz and can be realized in burst regimes [15,67,68,69,70]. Such a short interval between laser pulses can be achieved with special techniques of pulse dividing, such as crystals array or optical branches [28,71,72]. A more practical approach is usually to use long enough time delays when the plasma is gone upon the arrival of the next pulse.

Another alternative is application of a scanning strategy with special distribution of laser spots, where the distance between laser spots will be bigger than the plasma plume size (more than 200–300 µm). It can be multibeam surface processing with low frequency of laser pulses or high-speed surface scanning equidistant distribution of laser spots [9,73,74]. Several existing scanning techniques of distant laser spot distribution will be discussed in the Section 3 of this review. 

### 2.2. Heat Accumulation in Pulsed Laser Surface Processing

There are a lot of papers in which the important role of heat accumulation during laser surface processing was discussed [40,75,76,77]. It was shown that in some optimal conditions, heat accumulation becomes a positive factor for increasing the ablation rate or improving the quality of the machined surface [31,42,78,79,80,81,82,83,84]. However, undesired high heat accumulation leads to thermal surface degradation with material boiling, intense oxidation and uncontrollable splash formation [40,75,76,85,86,87]. The evaluation of the heat accumulation level can be performed by thermal field modelling or by experimental [32,76,86,88,89,90].

After ablation, a thin remelted layer with a number of point defects and dislocations, produced by ultrafast cooling and shock wave, remains in the laser spot area [91,92]. The output laser-irradiated surface will be contaminated by precipitation of the ablated products and associated oxidation of the upper surface layers [93,94,95,96]. In most cases, the surface ablation process has a semi-thermal character ($k_{B}T_{i}\geq \varepsilon_{b}$). For example, at Gaussian distribution of energy in the laser spot, the laser pulse residual heat stays in the subsurface layers and in the near-ablated zone. The residual heat in subsurface layers can be brought by secondary effects: heat conductivity from ablated layers, ballistic and diffusion effects, convective and radiating energy exchange between the ambient and solid target, and shock wave spreading [37,49,93,97,98]. These residual effects have a great influence on the quality and efficiency of LST in a high-repetition multi-pulse regime [31,76,99]. Of course, even a single laser pulse can produce many thermal effects: material boiling, oxidation and splash formation. These thermal effects have short-term character, and in the biggest cases, high-temperature fields dissipate within 2–5 µs [41,76]. However, in the case of multi-pulse laser surface processing, the residual heat is rising from pulse to pulse in the laser-affected zone. Such heat accumulation is able to prolong and intensify the thermal processes to an undesired level, although this occurs in the case whereby one single pulse is unable to initiate significant thermal processes.

The value of heat accumulation can be predicted theoretically as a sum of the residual heat from all laser pulses in a laser pulse sequence [40,41]:

(3) $$T\left(r,t\right)=\sum_{i=0}^{N_{f}}∆T_{i}\left(r,\;t+i\cdot \frac{1}{f_{pulse}}\right)$$

where $T\left(r,t\right)$ is the temperature in the point r at the time moment t, $N_{f}$ is the full number of the laser pulses in the pulse sequence, ${∆T}_{i}$ is the residual temperature after i-th laser pulse and $f_{pulse}$ is the laser pulse generation frequency. In this equation, the residual temperature ${∆T}_{i}$ after absorption of a discrete laser pulse can be approximately defined from a 3D model with an instance heat point source [41,100,101]. A more detailed study of the heat accumulation effects under moving Gaussian laser spots was conducted by Bauer et al. [40]. For evaluation of heat accumulation under a laser-scanned surface in a fixed subsurface point, a semi-planar thermal model can be applied [76,102]:

(4) $$∆T\left(r,t\right)=\frac{F\cdot e^{-\frac{2r_{x}^{2}}{w_{0}^{2}}}}{\rho \cdot c\cdot \sqrt{4\cdot \pi \cdot \alpha \cdot t}}$$

where x is the coordinate of a fixed surface point, $r_{x}$ is the distance from the centre of the laser spot to the fixed surface point x, α is the thermal diffusivity, ρ is the material density and c is the specific heat. It has been found that the maximal heat accumulation in a thin subsurface layer is achieved within a specific time interval when the laser beam central point has already passed the fixed subsurface point [76,102]. In the exemplary work of F. Bauer et al. [40], the critical temperature for heat accumulation was defined through experiments involving offset temperature shifting in the laser-scanned surface. The defined offset temperature shift was compared with the corresponding temperature shift in thermal simulations. It was shown that the critical temperature for heat accumulation in steel surface processing has a value near 607 °C. Oxidation and surface degradation were detected at higher temperatures of the scanned surface preheating, even when other laser scanning parameters were not changed [40,76].

There are several other approximation methods for the evaluation of the heat accumulation level, for example, post-control of laser-processed surface by microscopy and profilometry [11,40,103,104,105,106,107] or energy-dispersive X-ray spectroscopy (EDX) of oxidation level [11,40,41,84,108]. It gives us the possibility of defining some limitations of the applied scanning strategies, but such methods are not able to determine temperature changes, which appear during the laser surface processing.

For the direct control of temperature changes under laser beam scanning, a contact method of temperature changes can be applied [68,85]. In this case, the measurements are not affected by optical effects, such as emissivity changes, plasma shielding or undesired influence of laser beam reflection on optical measurements. The disadvantages of such methods are the volumetric character of the achieved data and long response time of the measurements. 

**Figure 2:**
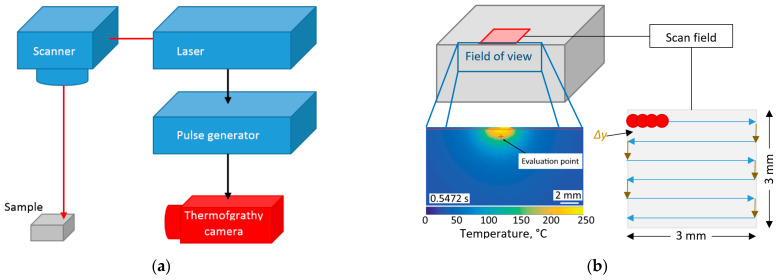
Heat accumulation detection with IR camera: (**a**) IR detection with thermal camera of heat accumulation in laser surface scanning process; (**b**) temperature distribution on side surface under scanning field [86].

Non-contact distant detection of temperature changes can be performed via the application of thermal IR cameras [86]. The application of an IR camera gives a mean value of temperature changes (maximal frame rate 1–2 kHz only [109]), and it does not recognise thermal changes after every individual laser pulse (Figure 2). A similar technique was applied for the detection of heat accumulation during the direct laser interference patterning (DLIP) process, but the camera was installed above the surface [110]. Such a solution is useful for the detection of surface distribution and the long-term dynamic of laser-induced heat accumulation.

**Figure 3:**
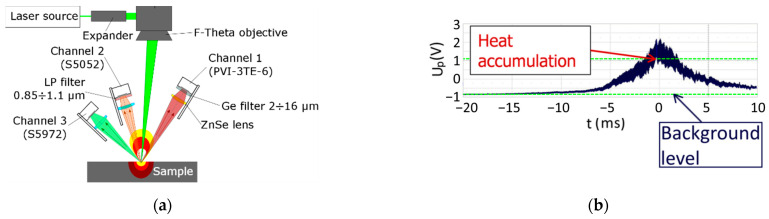
Fast detection of laser-induced surface heating: (**a**) IR measurement set-up with three IR photodiodes [102]; (**b**) evaluation of heat accumulation by subtraction algorithm [76].

For non-contact measurements with sub-nanosecond resolution and a frame rate reaching tens of GHz, the IR detectors can be applied [102,111,112] (Figure 3a). Such IR measurements are fast enough for detection of pulse-to-pulse heat accumulation, and they can be applied for comparing several types of LST: straight line grooving [76], surface micro-objects formation by different scanning strategies [113,114,115], LIPSS (laser-induced periodic surface structures) creation by laser multibeam processing [116] and other [117,118]. Such fast in-process IR measurements of heat accumulation were used for detecting thermal regimes during laser surface processing (Figure 3a) [76,112]. The heat accumulation was evaluated as a subtraction of the background level signal from the thermal IR signal (Figure 3b). In a similar way, an optimal regime of laser surface processing, LIPSS formation, phase changes or other laser-initiated effects can be evaluated [111,116,119,120,121]. The application of the multibeam technique decreases heat accumulation by dividing of thermal load into several separated fluxes, when instead of one gigantic laser pulse, the surface is irradiated by an array of laser spots with low energy [90,122].

In the case of LIPSS formation, an additional mechanism of heat conversion appears when the absorbed energy (after the laser pulse) is divided between different thermal processes, and it can affect the resulting heat accumulation. Such concurrent phases’ affection of heat accumulation during LIPSS formation was detected as a thermal double-maxima signature in fast IR-thermal measurements [116].

An interesting and original effect of the influence of temperature regime on LST processing and LIPSS formation was presented in the newest work by W. Gao et al. [123]. In this work, it was shown that decreasing the laser-processed surface temperature to below the freezing point has the potential to dramatically change the LST process. It is mentioned that the local frost layer around the laser-irradiated spot melts into water, helping to boost ablation efficiency, suppress the recast layer and reduce the heat-affected zone, while the remaining frost layer can prevent ablation debris from adhering to the target surface. The frost layer eliminates the debris deposition and recast layer, and LIPSS formation has a mechanism similar to high-spatial-frequency (HSF) LIPSS formation in water [124,125].

The newest results in experiments on the detection of heat accumulation have shown that the highest effectivity is achieved when the temperature of the surface reaches ~800 °C [76]. At the same time, it was shown that the most effective temperature regime does not lead to the best quality of surface processing, but it corresponds to a lower value of heat accumulation around 600 °C [40,76,83].

The correct choice of scanning strategy and optimization of laser beam parameters brings the possibility to overcome the aforementioned physical limitations, especially heat accumulation. The next development of the IR in-process fast measurements will be in on-fly control of the laser beam parameters during laser surface scanning. The on-fly IR control of the laser surface processing in combination with high-speed scanning technique is a way to achieve the high standards of Industry 4.0.

## 3. Scanning Techniques of LST

**Figure 4:**
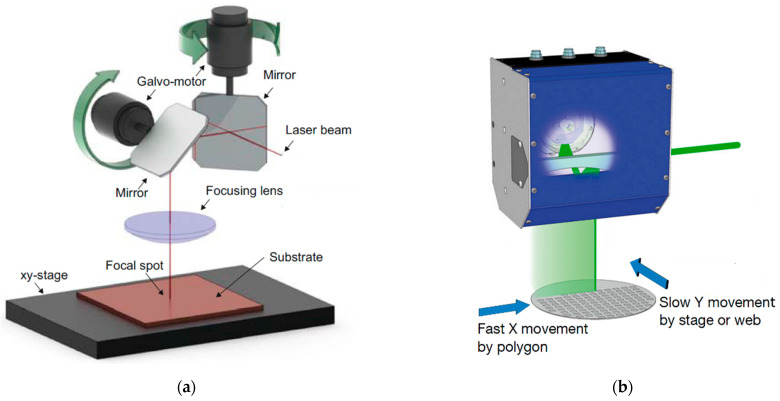
Inertial laser scanning systems: (**a**) galvanometer scanner; (**b**) polygon scanning technique (adapted from [126,127]).

The need for increasing the throughput of LST technologies stimulates the development of new strategies for high-speed laser surface processing. There are several well-known scanning technologies for high-speed laser beam deflection: galvo scanners, polygon scanners, piezo scanners, static and resonant scanners, micro-lens scanners, electro-optic deflectors (EOD) and acousto-optic deflectors (AOD) [21,103,126]. The inertial scanning systems have a maximal deflection angle and a number of resolvable spots on the scanned surface [126]. There are two traditional techniques for high-speed laser surface machining with a large processing area: galvanometer beam scanning and polygon optical scanning systems (Figure 4) [127,128]. The maximal scanning speed of the available galvanometer scanners lies in the range of 10–40 m/s, whereas the polygon scanner is able to achieve a scanning speed of more than 1000 m/s [127,129,130]. The higher speed of the polygon scanners is of great benefit in the fast provision of LST in large areas. However, polygon scanners do not provide the smooth wall profiles of vector scans for cutting a circumference or trepanning large holes greater than 50–150 µm. The laser beam deflection in polygon scanners should be corrected by an additional galvanometer scanner. For LST, when the processed area is smaller than 15% of the working field, the polygon scanners are not cost-efficient, and alternative techniques will be more suitable [127]. The correct choice of scanning strategy helps to improve the laser-processed surface quality and precision of the laser pulse delivery.

The output processing rate of LST depends on a favourable choice of the combination of the scanning strategy with different scanning techniques (Table 1, rows 1 and 2). The maximal processing rate of 9000 cm^2^/min for DLIP was found by a team from Fraunhofer Institute IWS [131,132,133,134]. The period and distribution of laser surface textured objects with DLIP are directly dependent on the wavelength of the laser beam [135]. This limitation makes it difficult to apply the DLIP for texturing surfaces in cases of irregular structure or complex nonsymmetrical textures, for example, for super hydrophilic surfaces, high optical absorbance and hydrodynamic effects. The process of the formation of such a structure is based on self-organized effects, cone-like structure formation or programmable direct laser machining with spot size resolution [136,137,138]. For the laser-controlled formation of fine complex submicron structures, a combination of the DLIP technique with dynamic systems has been provided. The processing rate of such a technique is about 0.7–10 cm^2^/min [132,139]. The benefit of such a combination of DLIP with regular micro-texturing techniques gives the possibility to create unique hierarchical structures [132,140].

Submicron surface structures might be created by laser scanning with the self-organized formation of LIPSS [116,141]. In this case, the laser scanning parameters, such as laser spot overlapping and scanning speed, have a key role for highly regular LIPSS (Table 1, rows 3 and 16). The achieved processing rate for LIPSS formation directly depends on the applied scanning speed. For example, I. Gnilitskyi et al. [142] have reported a processing rate of LIPSS forming on stainless steel equal to 6.3 cm^2^/min with a scanning speed of 3 m/s. The last study predicts several times higher processing rates with polygon scanners [77]. LIPSS forming is competitive with industrial standards of nano-manufacturing (~1 cm^2^ in 10 s) [143]. Like the DILP technique, LIPSS might be applied for the formation of hierarchical surface structures in combination with micro-texturing. The benefit of such a solution is that the period of the upper LIPSS can be smaller than half of the laser wavelength [143].

Another highly productive LST strategy is via forming an array of micro-objects by dividing the laser beam with diffraction or shadow masks [144,145,146,147] (Table 1, rows 12 and 17). This scanning strategy could be used in cases when the pulse energy is high enough to be divided into multi-beams [90]. The distance between laser spots is given by mask parameters or diffraction light distribution. In the case of the application of a solid-state static mask, the spot distance is constant, and part of the laser beam energy will be lost. The application of spatial light modulators (SLMs) gives us the possibility to change the laser spot distribution of the scanning process, but the average power will be limited to under 300 W [148]. However, a processing rate of up to 1800–5400 cm^2^/min in a multibeam scanning solution can be achieved [148,149].

LST by straight hatch lines is the most suitable strategy for the polygon scanner technology (Table 1, rows 13–16, 20, 22). In this case, the laser beam scanning speed achieves high values, up to 800–2000 m/s [42]. The polygon scanner has a throughput several times higher in comparison to galvanometer scanners [150]. However, providing LST with a polygon scanner needs to involve a correlation between mirror position and laser pulse generation for the precise formation of micro-objects. This imposes a restriction on the scanning speed for LST. The maximal processing rates up to 7680 cm^2^/min were achieved for a linear texture, and this was performed at 320 m/s [77]. However, for polygon scanners, the problem of processing arrays of micro-objects with specific geometry remains unresolved [151]. It is difficult to control the laser drilling of micro-objects with high-speed scanning, because there are substantial data in relation to large arrays with small objects or micro-objects, up to 800 MB per second [130]. Additionally, there is not enough time for precise control of laser spot distribution inside every micro-object in the array. Moreover, ultra-high-speed laser beam processing with polygon scanning involves artefacts such as jitter, banding, bow and other problems characteristic of these systems. These artefacts involve two components: periodical and random. There are several hardware techniques for reducing polygon scanner artefacts, but known classic methods of laser beam processing of the array of objects in ultra-fast scanning systems do not have a fully finished solution to the mentioned problems, and scanning techniques must still be improved [151,152].

The galvanometer scanner can create curved lines purposefully with the fast swinging of two deflection mirrors (Figure 4a). This technique was applied in direct laser formation of an array of micro-objects with different structures (Table 1, rows 6–11). The galvanometer scanner is able to achieve a processing rate of up to 428 cm^2^/min for forming an array of micro-objects with a one-beam simple scanning technique at one pulse per object [153]. The high precision formation of surface structures with hatch filling of more complex micro-objects reduces the processing rate to 25 cm^2^/min [38]. Noticeably higher processing rates with galvanometer scanners might be achieved by splitting the laser beam into several spots. In this case, a processing rate of up to 5400 cm^2^/min is reached [148]. A multibeam solution has potential for industrial applications, especially where there is a need to create a wide array of periodical surface microstructures [116,148].

**Table 1:** The surface processing rate of different scanning strategies.

Scanning Strategy		Scanning Technique	Structure Period (µm)	Processing Rate (cm^2^/min)	Scanning Speed (m/s)	Reference
1	DLIP-head (ps-laser)	Sample movement	0.343–1.064	100–9000	1	[131,132,133,134]
2	DLIP-head (ps-laser)	Galvanometer scanner	0.532–1.064	0.7–10	16·10^−3^–6.8	[132,139]
3	LIPSS (fs-laser)	Galvanometer scanner	0.9	6.3	3	[142]
4	Path cutting (fs-laser)	Sample movement	0.8	0.01	0.3	[154]
5	Unidirectional scan (ps-laser)	Sample rotation and acousto-optic beam deflection	250	~46.8	1.5 (rotation) 40 (AOD scanning)	[155]
6	Hatch filling (ns-laser)	Galvanometer scanner	12.5–200	1.8–428	0.25–4	[153]
7	Point-by-point ablation (fs-laser)	Galvanometer scanner	30–40	0.4–0.75	~0.05–0.2	[156,157]
8	Hatch filling (ps-laser)	Galvanometer scanner	2000	0.15–0.20	0.5	[158]
9	Path writing (0.1 μs laser)	Galvanometer scanner	50	1.2	0.4–2	[159]
10	Hatch filling (fs-laser)	Galvanometer scanner	4	8–25	4.5–17.1	[38]
11	Interlaced (ps-laser)	Galvanometer scanner	1.2–6	0.017–2	0.024–0.6	[160]
12	Hatch filling (ps-laser)	Multibeam galvanometer scanner	500	5400	20	[148]
13	Hatch filling	Polygon scanner	14.5–40	148–7680	60–800	[77,127,129]
14	Hatch filling (ps-laser)	Polygon scanner	10	840	10–200	[42]
15	Hatch filling (fs-laser)	Polygon scanner	1–12	0.03, approx. 60	25	[161]
16	Hatch filling (fs, ps-laser)	Polygon scanner	40	43	15	[162]
17	Laser pulse pattern	Sample movement with mask	20	1800	–	[149,163]
18	Shifted path (ps-laser)	Galvanometer scanner	200	17.4	8	[102,164]
19	Shifted burst (ps-laser)	Galvanometer scanner	60–570	160	8	[102,113]
20	Unidirectional hatch	Polygon scanner and self-organizing	≲0.5	1510	560	[165]
21	Hatch filling with multibeam	Galvanometer scanner with DOE	~0.4	1910	9	[116]
22	Hatch filling with ns-laser	Polygon scanner	50	1386	200	[166]

## 4. Scanning Strategies

### 4.1. Classic Methods of Laser Beam Scanning

**Figure 5:**
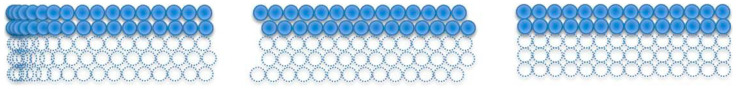

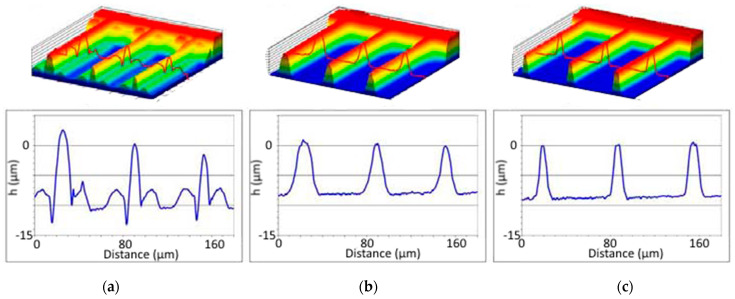
Precision and quality of bar texturing (from top to bottom: laser spots disposition, 3D and section profilometry): (**a**) without sky-writing; (**b**) with sky-writing; (**c**) synchronized system (10 ps, diameter 5.7 µm, 120 mW, 300 kHz, 1 µm pitch, 60 layers) [10,11].

In the classic methods, the precision of laser surface machining is reached by continuous control of the laser beam movement. Mirrors’ inertia in galvanometer scan heads requires additional time for acceleration and deceleration. Incorrect delays in laser switching on and off lead to floating of the overlapping at the edges of the scanning paths (Figure 5a). Corrections on the laser path edges are provided by sky-writing or by additional synchronization between laser mirror position and laser pulse generation. A strong correlation between laser pulse delivery and scanning mirrors’ position improves the precision of LST close to 1–2 µm [103,129] (Figure 5b,c). On the other hand, every additional correction of the scanning parameters may lead to an escalation of the processing time up to 50% [129,167].

**Figure 6:**
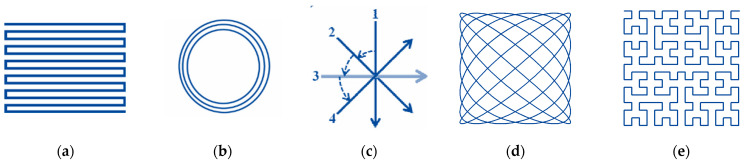
Scanning strategies for laser surface machining with different laser beam movement arrangements (adapted from references): (**a**) straight line hatching [177,178,179], (**b**) path filling [172], (**c**) angular hatching [175], (**d**) filling by Lissajous carves [176], (**e**) filling by Hilbert curves hatching [178].

The quality of laser surface machining also depends on the applied laser beam paths’ arrangement [161,168,169]. There are several main scanning strategies for filling the laser-textured objects with laser spots: straight hatching, path filling, interlaced filling, criss-cross texturing, unidirectional or bidirectional scanning, angular hatching, wobble scanning and their combinations [103,108,132,160,170,171,172,173,174,175,176,177] (Figure 6).

**Figure 7:**
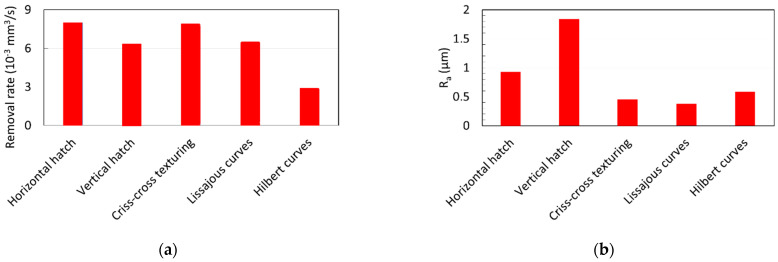
Evaluation of different hatch geometries: (**a**) matter removal rate; (**b**) average roughness analysis (4.5 W, 800 kHz, τ_p_ = 10 ps, diameter 34 µm, 0.63 J/cm^2^, hatch overlap 1.7 µm, 25 scans. Adapted from [176]).

The correct choice of scanning method significantly affects the efficiency and quality of laser material processing. Dold [176] has provided a detailed study of the influence of the different scanning strategies on the ablation rate, roughness and processing time of the laser surface machining (Figure 7).

It was shown that the highest efficiency of laser surface processing with scanning by straight hatching lines has an efficiency more than two times higher in comparison to fractal filling. The quality of a laser-processed surface depends not just on the laser beam filling strategy, but on the direction of the scanned lines, i.e., whether it is in a vertical or horizontal direction. This distinguishing feature of horizontal and vertical directions can be explained by the difference in the dynamics of X-scanning and Y-scanning galvanometer mirrors. 

All of the presented scanning strategies have their advantages and disadvantages. A discussion of a full long list of their variants and combinations would be inefficient. In the next part of this paper, the scanning strategies, which are widely used in the experimental realizations of LST, are discussed. Additionally, some special scanning strategies are discussed, which were developed for overcoming undesired heat accumulation or for achieving special conditions for LIPSS formation. 

### 4.2. Classic Strategies of Micro-Object Formation in LST

**Figure 8:**
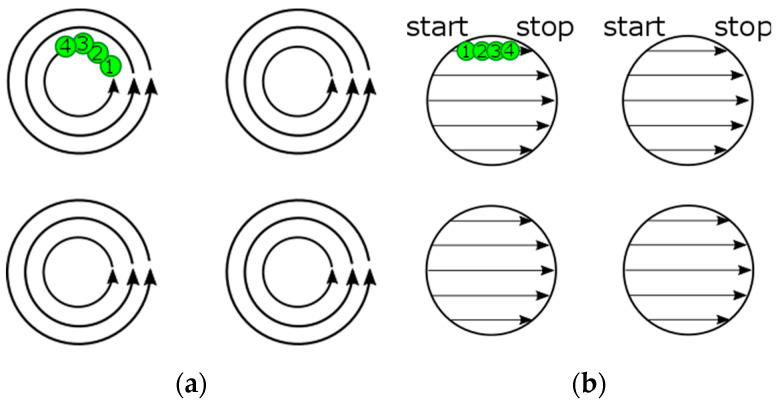
Laser pulse distribution for classic scanning strategies for laser writing of micro-objects: (**a**) path filling; (**b**) hatching. Numbers 1–4 show successive laser pulses.

In classic LST, generally, there are two most known scanning strategies of micro-object formation: path and hatch filling (Table 1). The first classic-path filling strategy of a micro-object array formation is similar to helical scanning with several concentric circles (Figure 8a). The dimple texturing is performed consecutively, as every next dimple in the array is formed only after all the filling paths inside the previous dimple have been completely finished. The short length of the laser beam paths inside every micro-object can increase the interline heat accumulation in addition to the inline heat accumulation.

The second classic strategy is hatch scanning of micro-objects in an LST array. The micro-objects on the textured surface are formed by straight scanning of the laser beam through all the micro-objects in one row (Figure 8b). This means that one hatching line belongs to the several dimples in the scanned LST array in one horizontal direction. The next hatching line is started only after fully finishing the writing of the previous hatching line in a row. Inline laser pulse generation should be stopped after writing one hatching segment and started again on the next dimple hatching segment.

In both these classic strategies, the processing rate $v_{PR}$ is decreased by “on-fly” synchronization between mirrors’ position and laser pulse generation. In the case of the classic path filling method, the inertia of the galvanometer mirrors becomes a principal limitation of scanning speed [180]. The application of the classic LST methods with high repetition rate lasers is additionally limited by physical effects, such as heat accumulation, plasma shielding effects and non-effective laser spots distance. Moreover, there are some technical limitations of the application of the classic LST methods at high scanning speed: low precision and a large amount of data needing to be processed in a short time [103,130,180].

### 4.3. Interlaced Method of Laser Beam Scanning

**Figure 9:**
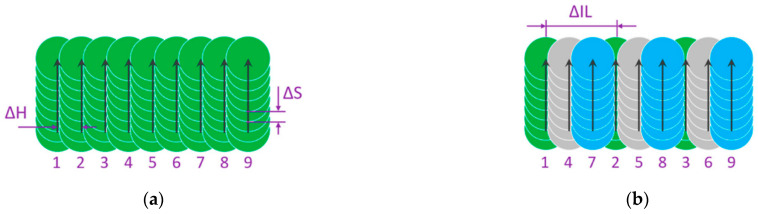
Straight line laser scanning strategies: (**a**) classic unidirectional sequential; (**b**) unidirectional interlaced. Meaning of symbols: ΔH—hatch distance; ΔS—distance between pulses in the laser beam scan direction; ΔIL—interlace distance; Numbers 1–9 show successive laser paths [160].

The distance between hatching lines and their ordering becomes important in the case of laser machining of temperature sensitive materials, for example, in selective laser melting (SLM) technology, composite materials treatment and the formation of biocompatible structures [25,41,181,182,183]. Heat accumulation between inline laser spots is not the only aspect responsible for thermally damaged results [184,185]. There are several types of heat accumulation leading to material damage: pulse-to-pulse-, rerun-, and geometry-density heat accumulation [14,186,187]. Overcoming the heat accumulation at high repetition rates and high speeds of laser surface processing can be achieved with the interlaced method. In this interlaced method, the scanning lines do not have sequential ordering (Figure 9) [108,122,160,188,189]. The application of the interlaced scanning method is able to improve the ablation rate from 4 up to 13 times in comparison to the classic sequential method (Figure 9a) [160].

**Figure 10:**
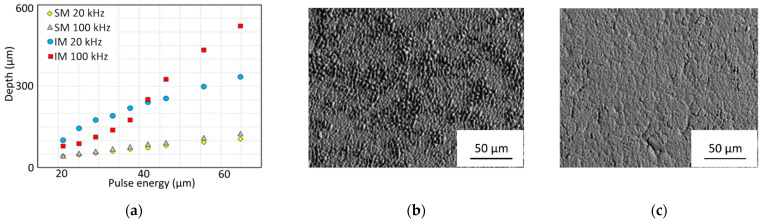
Application of the interlaced scanning method with a polygon scanner: (**a**) increasing the ablation rate above four times as compared to the classic sequential method (adapted from [160]); (**b**) surface machined with a pitch 4.9 µm with the sequential method; (**c**) surface machined with a pitch 9.8 µm with four interlaced patterns (reproduced from [108], with the permission of AIP Publishing).

The interlaced method of laser beam surface scanning of stainless steel was studied by Neuenschwander et al. [108]. With the interlaced method, the time interval between overlapping scanning lines is not equal to the period of the laser lines scanning, but it is given by the full scanning time of one area. The classic sequential surface scanning method initiates heat accumulation, and the processed surface is damaged by cavity formation (Figure 10b). Unlike the classic method, a surface machined via the interlaced method with the same spot distance between two neighbouring spots shows a good surface quality (Figure 10c).

It can be concluded that the interlaced method has great potential for laser surface processing with high repetition rates of several tens of MHz and high scanning speeds. For overcoming heat accumulation, the lateral distribution of the laser spots should be comparable to the laser spot diameter. It requires great scanning speeds, i.e., several hundred meters per second. At such scanning speeds, laser switching for controllable pulse picking will definitely not be possible anymore [108,173].

### 4.4. Shifted Laser Surface Texturing

**Figure 11:**
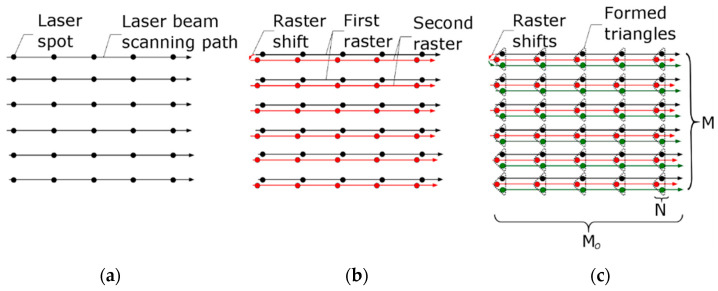
The shifted LST method of triangle array formation: (**a**) linear raster with one spot per one micro-object location; (**b**) one small shift in the linear raster to the next position; (**c**) formation of triangular objects in an array by two sequenced raster shifts.

The physical and technical limitations of the classic and interlaced strategies [160,173] can be overcome by using an asynchronous surface scanning method. Shifted laser surface texturing (sLST) is an asynchronous scanning strategy which was developed for faster laser writing of a wide array of repeating micro-objects [114,164]. The algorithm of sLST can be explained in an example with an LST array of triangles. Scanning is performed on straight lines, and asynchronous laser pulsing has a continuous character during the processing of the whole scanning line. In this approach, the laser pulses are rapidly rasterized on the whole processed surface by applying only one laser spot per one micro-object in the array (Figure 11a). The scanning raster is slightly shifted on the surface at every next application of the raster (Figure 11b). The sequence of shifts along a triangular shape produces an array of triangular micro-objects (Figure 11c).

**Figure 12:**
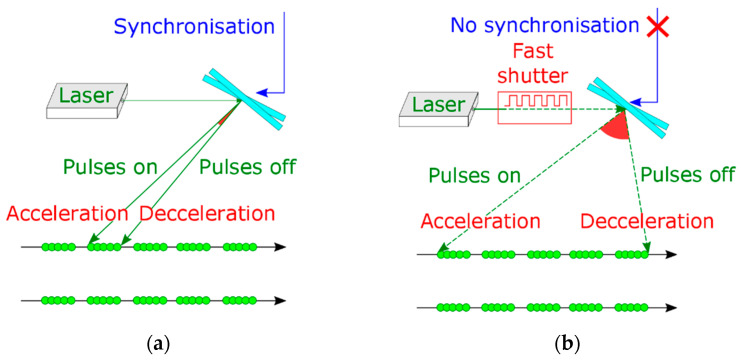
Equidistant straight segments formed by: (**a**) classic hatch strategy, in which it is necessary to control the mirror position and laser switching for every object in the array; (**b**) shifted LST in burst regime, in which there is no need to know the position of every object. Synchronization between laser and mirrors is provided only at the start and finish positions on the scanning field [113].

In the case of larger objects forming, a longer series of laser pulses can be applied instead of pulse-by-pulse laser surface processing. In this case, one laser scan raster contains an area of straight line segments (Figure 12). The classical strategies of LST need to have continuous synchronization between mirrors and laser switching for every segment in the scanned line (Figure 12a). Unlike this, in the asynchronous burst sLST method, the position of the straight segments is determined indirectly by the scanning speed of the laser beam and by the period between the bursts (Figure 12b). The form of the laser-written micro-objects is defined by the sequence of shifts in the linear rasters, which is similar to the one-spot-per-one-object strategy sLST (Figure 11) [114,164]. K. Ratautas et al. [190] have shown that the shifted method of laser spot distribution decreases heat accumulation several times and that such a method is suitable for high-temperature-sensitive materials.

It was shown by M. Gafner, S. M. Remund et al. [122] that the synchronized scanning method is able to achieve a higher precision at the cost of scanning speed. Higher scanning speeds with high precision can be achieved via the simultaneous application of the synchronized mirrors’ movement on raster line ends with laser spot distribution according to the shifted method (Figure 12b) [102,113,122,190]. However, in this case, feedback communication between the laser source and scanning system for every laser spot position puts a limit on the maximal scanning speed. The novelty of the sLST method lies in the combination of two principal properties of the laser scanning system: the inertia of the deflection mirrors and the stable frequency of the laser pulse generator. In this method, the laser works as an asynchronous source, and correction of the first laser spot position is needed only at the starting position of the scanning mirror.

### 4.5. Scanning Strategies Combined with LIPSS Formation

**Figure 13:**
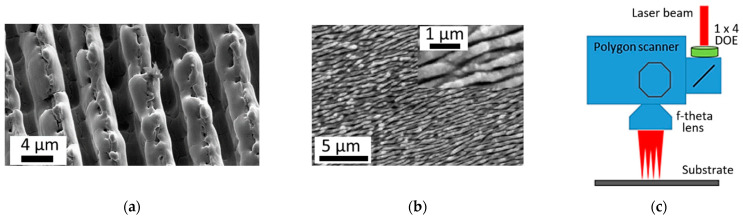
Fast laser surface processing: (**a**) close to the optical limit (bidirectional hatch) [168], (**b**) self-organized nanostructure [195], (**c**) multi-beam high-speed scanning technology [165].

Laser surface processing with application wave-optical effects such as multibeam interference and diffraction beam modulation in LST gives us the possibility of achieving a resolution at a level of detail close to the optical limit (Figure 13a) [73,132,133,140,191,192]. DLIP has provided one of the best results, showing the possibility to form a surface pattern with 180 nm detailing. It is lower than the wavelength of the applied laser (266 nm), and it is already very close to the theoretical Abbe optical limitation of λ/2 [193]. Through the application of immersion techniques, detailing of features with a size down to λ/4n is possible, where n is the refractive index of the surrounding medium [194].

The application of laser-initiated self-organized processes allows us to overcome the wavelength principal limit by achieving surface nano-structuring and a remarkable increase in the processing rate (Figure 13b) [116,165,195,196,197]. The combination of wave-optical effects with one of the known laser surface scanning techniques has great potential for the application of LST on a wide surface area (Figure 13c) [165,198,199]. For example, the papers of Lasagni et al. [131,132,133,134,200] introduced a combination of hatch-scanning techniques and interference patterning, where the processing rate achieved 0.9 m^2^/min. A higher processing rate and smaller period of processed relief can be achieved in experiments on surface texturing by forming self-organized LIPSS structures [195,201]. Schiele et al. reported the achievement of a processing rate up to 1.5 m^2^/min with self-organized LIPSS nanostructures on metallic surfaces [42,165].

The application of a multispot technique in combination with scanning technology is a helpful method for decreasing the thermal load on material with an expanding processing area [122,165]. There are two main methods of multibeam forming: Diffractive Optic Element (DOE) and Spatial Light Modulation (SLM) [202]. In the case of the application of DOE, the periodicity of multibeam spot distribution is fixed, and the flexibility of an applied laser scanning strategy becomes limited by diffraction spot distribution [73,122]. On the other hand, SLM units are able to change laser spot distribution during scanning processes with a frame rate of 60–180 Hz [202,203]. However, the application of SLM units has limitations if the maximal laser beam power is above 200 W [148]. Improving the existing scanning strategies with LIPSS and their combination with multibeam technology looks like the most promising way for reaching industrial scales of LST [116,122,165,204].

## 5. Conclusions

In this paper, classical and recently developed scanning strategies utilizing laser surface texturing (LST) were compared. In the beginning, plasma and particle shielding effects and heat accumulation were described as basic physical limitations of short and ultrashort LST. Different methods of heat accumulation evaluation for the optimization of scanning strategies were briefly discussed. Several methods of laser beam movement arrangements contained in the scanning strategies were discussed, such as straight hatching, path filing, criss-cross texturing, and Lissajous or Hilbert curves filling. The main attention was paid to the classical path and straight hatch filling strategies coupled with galvanometer and polygon scanning techniques as the most known strategies. It was observed that classical scanning strategies of LST are limited in terms of processing rate by the mentioned physical limitations. Alternative, recently developed scanning strategies with high-processing-rate LST were discussed, namely, the interlaced method and shifted LST. It was shown that a combination of several techniques, such as multibeam processing, asynchronous shifted LST strategy and LIPSS formation, can offer a way to achieve a higher processing rate in LST. The next step in achieving the high standards of Industry 4.0 can be the application of the on-fly non-contact IR control of the temperature regime in laser surface processing coupled with a high-speed scanning technique.

## Data Availability

No new data were created or analyzed in this study. Data sharing is not applicable to this article.
